# Harnessing Graphene-Modified Electrode Sensitivity for Enhanced Ciprofloxacin Detection

**DOI:** 10.3390/ijms25073691

**Published:** 2024-03-26

**Authors:** Lidia Mǎgeruşan, Florina Pogǎcean, Bogdan-Ionuţ Cozar, Septimiu-Cassian Tripon, Stela Pruneanu

**Affiliations:** National Institute for Research and Development of Isotopic and Molecular Technologies, Donat Street, 67-103 Cluj-Napoca, Romania; florina.pogacean@itim-cj.ro (F.P.); bogdan.cozar@itim-cj.ro (B.-I.C.); septimiu.tripon@itim-cj.ro (S.-C.T.)

**Keywords:** ciprofloxacin detection, graphene modified electrodes, cyclic voltammetry, linear sweep voltammetry, chronoamperometry

## Abstract

Increased evidence has documented a direct association between Ciprofloxacin (CFX) intake and significant disruption to the normal functions of connective tissues, leading to severe health conditions (such as tendonitis, tendon rupture and retinal detachment). Additionally, CFX is recognized as a potential emerging pollutant, as it seems to impact both animal and human food chains, resulting in severe health implications. Consequently, there is a compelling need for the precise, swift and selective detection of this fluoroquinolone-class antibiotic. Herein, we present a novel graphene-based electrochemical sensor designed for Ciprofloxacin (CFX) detection and discuss its practical utility. The graphene material was synthesized using a relatively straightforward and cost-effective approach involving the electrochemical exfoliation of graphite, through a pulsing current, in 0.05 M sodium sulphate (Na_2_SO_4_), 0.05 M boric acid (H_3_BO_3_) and 0.05 M sodium chloride (NaCl) solution. The resulting material underwent systematic characterization using scanning electron microscopy/energy dispersive X-ray analysis, X-ray powder diffraction and Raman spectroscopy. Subsequently, it was employed in the fabrication of modified glassy carbon surfaces (EGr/GC). Linear Sweep Voltammetry studies revealed that CFX experiences an irreversible oxidation process on the sensor surface at approximately 1.05 V. Under optimal conditions, the limit of quantification was found to be 0.33 × 10^−8^ M, with a corresponding limit of detection of 0.1 × 10^−8^ M. Additionally, the developed sensor’s practical suitability was assessed using commercially available pharmaceutical products.

## 1. Introduction

Ciprofloxacin (CFX) is a synthetic broad-spectrum antibiotic that is a member of the fluoroquinolones antibiotic class [1], prescribed in the treatment of numerous bacterial infections, including urinary and respiratory tract; skin, soft tissue, joint and bone; eye and ear; sexually transmitted; and all sort of infections that other antibiotics have been unable to treat [2,3]. It is administrated to prevent meningitis in persons who have had close contact with someone infected with this disease and it is also employed to treat individuals who have been exposed to anthrax [4] or some forms of plague [5]. Ciprofloxacin has additionally caught the scientific community’s interest due to its apoptotic and anti-proliferative actions in numerous cancer lines [1]. Normally, at therapeutic levels, adverse health effects are minor and largely related to gastrointestinal disturbances such as nausea and diarrhoea. However, it was reported that CFX intake can also lead to severe allergic reaction; photosensitivity; tendon difficulties (pain, tearing or swelling); organ and nerve damage; severe mood or behavioural disorders; cardiac problems; and dangerously low blood sugar. Growing research suggests that it may cause unexpectedly severe liver damage [6,7,8,9]. Being one of the most effective drugs against both gram-negative and gram-positive bacteria, CFX is extensively employed both in human and veterinary sectors. More than 75% of the ingested CFX is excreted as waste product in its un-metabolized form and reaches the environment [10], contributing to antibiotic resistance or entering both human and animal food chains. Thus, it is no surprise that CFX was reported to appear as residue in dietary items originating from animals, causing a variety of negative consequences for the consumers including immunotoxicity, genotoxicity, hypersensitivity, carcinogenicity and hormone disorders [11].

In this general context, the rapid and sensitive detection of CFX is of great interest. Various analytical techniques for its detection have been reported, the most common involving spectroscopic (e.g., UV–Vis spectrophotometry [12,13] and fluorescence spectroscopy [14,15]) or chromatographic methods (high-performance liquid chromatography [16,17,18], as well as capillary electrophoresis [19]). The major disadvantages of all these so-called traditional tools include the relatively long analysis times and the necessity of well-trained personnel to operate the high-cost equipment. Among the different methods, the electrochemical approach manages to overcome all the above-mentioned inconveniences and to offer a relatively rapid, simple, highly sensitive and economically advantageous alternative [20,21].

A variety of electrodes have previously been developed and employed in CFX detection. Al-Ghamdi and Bani-Yaseen [22] used a mercury electrode; a boron–diamond electrode was employed by Garbellini et al. [23]; while a screen-printed diamond electrode was employed by Matsunaga et al. [24]. Santos and his co-workers [25] developed an electrochemical protocol for simultaneous CFX and paracetamol detection; a similar protocol was reported by Pollap et al. [26] as well as Kergaravat and her co-workers [27]. The usage of carbon-based conductive materials (such as carbon nanotubes, graphene, graphene–polymer composites) has been shown to be extremely efficient [28]. A reduced graphene oxide/poly(phenol red) modified glassy carbon electrode was reported by Chauhan et al. [29]; Xie et al. [30] studied CFX detection based on a graphene-modified glassy carbon electrode; while Pan and his co-workers [31] used a graphene oxide-modified screen-printed electrode to detect CFX residue in milk. In the majority of these reports the acquired sensitivity and selectivity are frequently limited to the range of 0.1–0.5 µM.

The purpose of this work is to offer a new approach for CFX detection based on a glassy carbon electrode modified with graphene. Compared to previous reports, in this case the electrode material has the considerable advantage of being prepared using a relatively simple and ‘green’ technology, avoiding the usage of toxic organic solvents in the preparation process and reducing the reaction times and costs of employed chemicals. While previous studies may have explored graphene-based electrodes for the concomitant sensing of CFX and various other analytes [32], this paper focuses specifically on detecting CFX. Furthermore, the developed protocol likely demonstrates superior performance compared to existing methods, ensuring low analysis time; high measurement sensitivity and selectivity; and considerable sensor stability, repeatability and interference capacity. Overall, the originality of this research stems from its superior electrode performance and its specificity, detailed characterization and practical implications, and it can contribute towards advances in the field of electrochemical sensing with implications in various sectors, including healthcare, pharmaceuticals and environmental monitoring. This paper includes thorough experimental validation using real sample analysis to support its claims and a detailed discussion of mechanistic insights into the interactions between graphene-modified electrodes and CFX molecules, adding a deeper understanding of the detection mechanism, which can inform further research and developments in the field. Demonstrating the practical applicability and validation of the developed sensor in real-word scenarios strengthens the originality of the research by showcasing its potential for tangible impact and utility beyond laboratory conditions.

## 2. Results and Discussion

### 2.1. Morphological and Structural Characterization of Graphene

**Figure 1:**
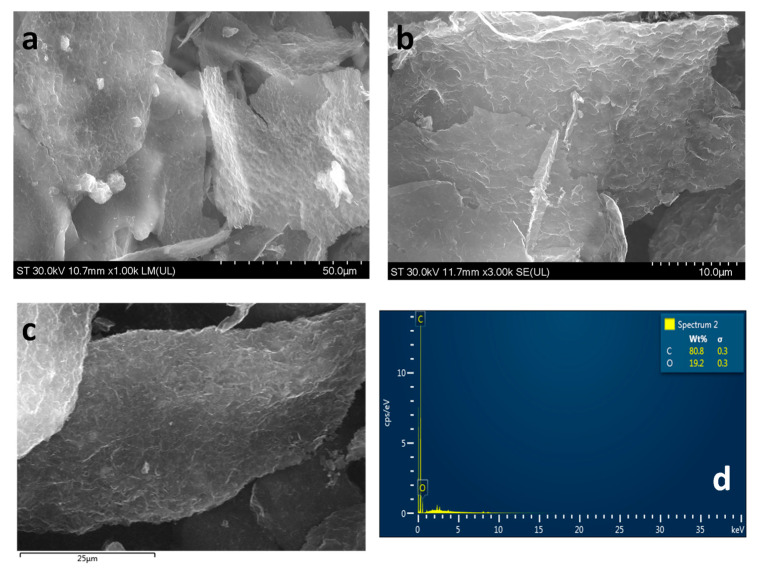
SEM micrographs of the investigated graphene sample (**a**–**c**); EDX spectrum indicating the presence of carbon and oxygen elements (**d**).

The surface morphology of the as-prepared material was investigated by SEM (Figure 1a,c). As can be seen, the sample contains large flakes in the order of tens of micrometres, with the typical wrinkled features on the surface due to the strong electrostatic repulsion between the two-dimensional confined layers. The large size of the flakes is an indication of the gentle exfoliation process which occurs in the selected electrolyte. This can be also associated with the lack of defects in the basal planes of the sheets, suggesting that the defects are mainly present at the sheet edges. EDX was employed to check the composition and sample purity. The EDX spectrum presented in Figure 1d displays the distribution of carbon and oxygen elements present at the surface.

**Figure 2:**
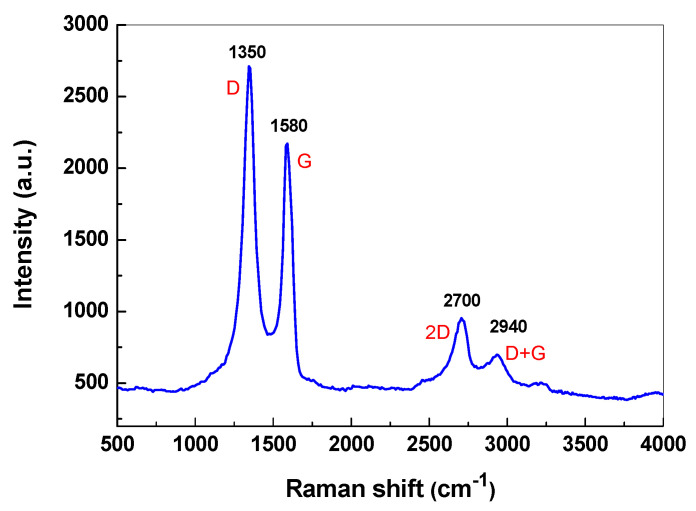
The Raman spectrum of the graphene sample, showing the presence of the main peaks of graphene: D, G, 2D and D+G.

Raman spectroscopy is a very useful technique which helps in characterizing the properties of graphene and graphene-based materials. The interaction between the monochromatic laser beam (λ = 532 nm) and the graphene sample takes place either through elastic (Rayleigh) or inelastic scattering, in which case a down-shift (Stokes) or up-shift (anti-Stokes) of the laser energy occurs [33]. In the prepared graphene sample (see Figure 2), two main peaks in the Raman spectrum can be observed: the defect—D (1350 cm^−1^)—and graphite—G (1580 cm^−1^)—peaks, both of them being in-plane vibrational modes, accompanied by the 2D peak (2690 cm^−1^) as a second-order overtone of the D peak. The D and 2D peak positions are dispersive; therefore, they are dependent on the laser excitation energy (2.33 eV). In addition, the D+G peak can be seen at 2940 cm^−1^, being a combination of scattering peaks [34]. The intensity of the D band is higher than that of the G band (*I_D_*/*I_G_* = 1.25), indicating the presence of some defects in the graphene lattice, which may help with the transfer of electrons from the solution to the electrode substrate. According to Cançado et al. [35] the in-plane crystallite size (*L_a_*) of graphene is related to the *I_D_*/*I_G_* ratio (see Equation (1); *E_l_* represents the laser excitation energy in eV), giving an indication of the magnitude of the defect-free domains. In this case, *L_a_* was found to be 15.2 nm.

(1) $$L_{a}(nm)=\frac{560}{E_{l}^{4}}{\left(\frac{I_{D}}{I_{G}}\right)}^{-1}$$

**Figure 3:**
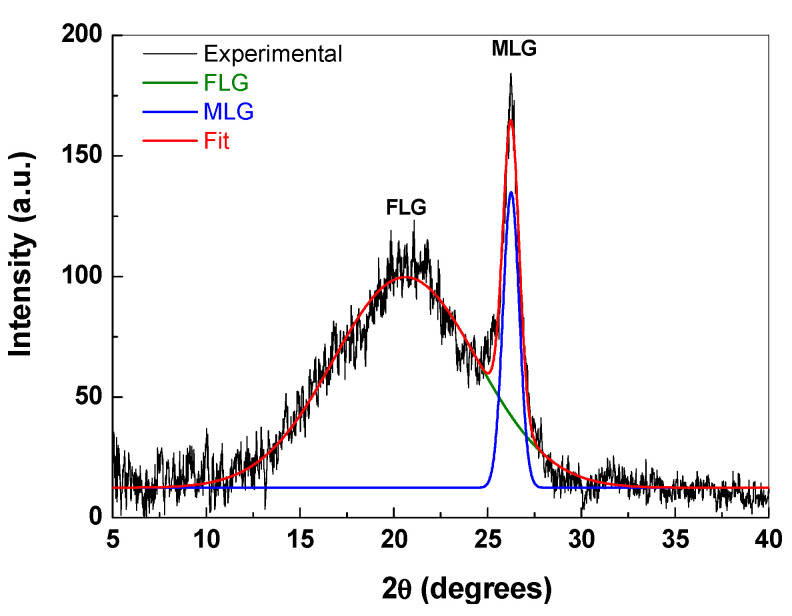
The XRD pattern of exfoliated the graphene sample (background subtracted pattern).

The XRD technique is another important tool employed to get information about some of the structural parameters of graphene samples, such as the mean size of graphene crystallites (*D*), the average number of layers (*n*) present within the crystallites and the interlayer distance (*d*) [36]—see Table 1. The recorded pattern is presented in Figure 3 and reveals two main peaks: the first one is small and broad and is attributed to the reflections of few-layer graphene (FLG; ~21°); the second one is narrow and of higher intensity and is attributed to multi-layer graphene (MLG; ~26°).

As expected, the identified structures have different values for the interlayer distance, such as 0.433 nm for FLG and 0.34 nm for MLG. The higher value observed for FLG may be due to a larger amount of oxygen-containing groups attached to the graphene layers. In the case of MLG, the number of functional groups/structural defects is smaller and so is the d value. The amount (%) of FLG and MLG present within the sample was also determined from the ratio of the corresponding peak area to the total area of the pattern. As can be seen in Table 1, FLG is predominant within the sample (86.4%, while MLG is 13.6%). In addition, the mean size of MLG crystallites is around 10 nm, indicating that large flakes may be formed due to π-π stacking interactions between graphene layers.

UV–Vis and FTIR spectra of the graphene sample are shown in Figure S1 of the Electronic Supplementary Materials. From the UV–Vis spectroscopic studies, it can inferred that the optical absorption of graphene is dominated by the π → π* transition of the aromatic C–C bonds, which generates a major band centred at about 268 nm. The position of this spectral feature is different from the one typically observed in graphite (232 nm) and is similar to the reports of Çiplak et al. [37] and Johra et al. [38]. The π-π* plasmon peak depends on two kinds of conjugative effect: one is related to nanometre-scale sp^2^ clusters and the other arises from linking chromophore units, such as C=C, C=O and C–O bonds [39].

The FTIR spectrum (Figure S1b) was used to identify the appearances of the main functional groups in the exfoliated graphene structure. A strong and broad O-H stretching vibration band can be observed at 3409 cm^−1^, together with the characteristic bands associated with the C=O stretching band at 1729 cm^−1^; the O-H deformation vibration band at 1394 cm^−1^; and the C-O stretching vibration at 1041 cm^−1^. The existence of two peaks at 2923.78 cm^−1^ and 2846 cm^−1^ confirms the existence of sp^3^ C-H bonding in the reported material. The position of the bands is similar to that in previous reports [40].

**Figure 4:**
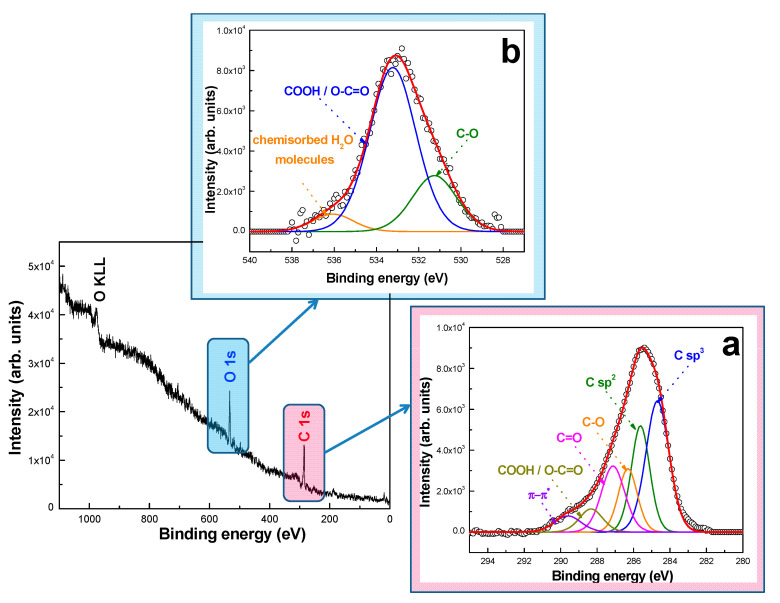
XPS characterization of exfoliated graphene and deconvolution of C1s (**a**) and O1s (**b**) levels.

XPS is a powerful surface-sensitive spectroscopic technique that can be used to determine the elemental compositions and chemical bonds of graphene derivatives. As visible in Figure 4, in the case of the EGr material survey spectra, there are two prominent peaks at the binding energies of 284 and 530 eV, commonly known as the C1s and O1s peaks. The deconvolution of these levels is presented in the insets of Figure 4. When it comes to graphene, there are two major features in the carbon spectra, corresponding to C atoms in a graphite-like sp^2^ hybridized state, representative of the carbon framework (both surface and bulk) and located at binding energies (BE) of 284.34 eV; and sp^3^ hybridized C atoms at 285.61 eV, indicating the presence of structural disorder which appears at the edges of the sp^2^ network in the form of carboxyl, epoxy, hydroxyl, ketones and quinones. The sp^2^ carbon fraction, which is influenced by the presence of functional groups, is the most critical parameter for determining the degree of oxidation. In the case of EGr material, the graphite exfoliation results in the modification of some original sp^2^ bonds of carbon atoms into sp^3^ bonds via bonding with oxygen, moving carbon atoms from their original sites to accommodate the off-plane sp^3^ bonds. Beside these main components, the deconvolution of the C 1s level implies the presence of three distinct peaks assigned to different carbon–oxygen bonded forms (C-O-C, C=O, O=C-O and COOH)—please see Table S1 of the Electronic Supplementary Materials. In the high binding energy region (and 289.61 eV), a small contribution assigned to the π→π* shake-up satellite band of graphitic carbons appears [41].

A clear piece of evidence of the presence of structural defects which determine the appearance of different functional groups at the surface of exfoliated graphene material is confirmed by the deconvolution of high-resolution O1s levels. According to this, the unsaturated carbon atoms can bind oxygen in the form of C–O, C=O and O–C=O functionalities (see inset b of Figure 4). Furthermore, a small contribution of about 6.61% was assigned to the adsorbed water molecules. Similar results were obtained for other exfoliated graphene samples prepared in our group [28,42,43,44]. Assignments of each deconvoluted XPS peak based on their binding energies (BE), atomic concentrations [AC, %] and full width at half maximum (FWHM) for each component are reported in Table S1 of the Electronic Supplementary Materials.

### 2.2. Electrochemical Studies

**Figure 5:**
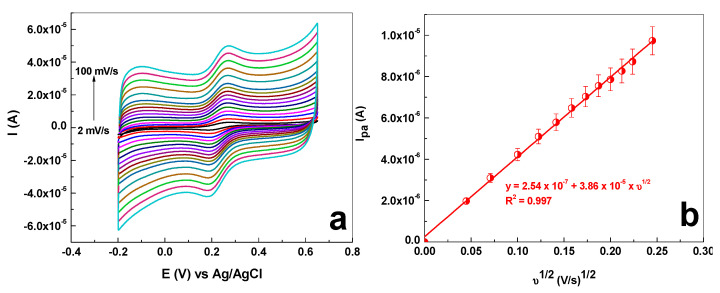
CVs recorded with EGr/GC at different scanning rates (2 to 100 mV/s) in solution containing 1 mM K_4_[Fe(CN)_6_] and 0.2 M KCl supporting electrolyte (**a**); the plot of I_pa_ versus υ^1/2^ and the corresponding linear regression equation (**b**).

In order to determine the active area of the graphene-modified electrode and to understand its electrocatalytic activity, CV measurements were recorded at different scan rates, from 2 to 100 mV/s, in a 1 mM K_4_[Fe(CN)_6_] redox indicator (0.2 M KCl supporting electrolyte—see Figure 5a). The anodic peak current intensity (I_pa_) was plotted versus the square root of the scan rate (υ^1/2^), then fitted using the following linear regression equation: I_pa_ = 2.54 × 10^−7^ + 3.86 × 10^−5^ × υ^1/2^ (R^2^ = 0.997), Figure 5b. The obtained active area (0.058 cm^2^) was calculated from the corresponding slope by employing the Randles–Ševčík equation [45]. Such a value is higher than that generally obtained for the bare GC electrode (0.028 cm^2^), demonstrating the successful immobilization and stability of graphene flakes on top of the electrode surface. In order to assess the surface modification reproducibility, five electrodes were modified with graphene and their active areas were calculated. The obtained values indicated that the differences between them were <10%, confirming the good reproducibility of the electrode surface modification.

In order to prove the advantage of using the graphene-modified electrode for electrochemical detection, Electrochemical Impedance Spectroscopy (EIS) was employed. This technique is very useful, since it can provide information about the charge transfer resistance (Rct) by applying a small sinusoidal signal (~5 mV) of various frequencies (0.1–105 Hz). In this case, we compared the EIS spectra (Nyquist plots) of bare and graphene-modified electrodes (see Figure S2 and the corresponding inset from the Electronic Supplementary Materials). As expected, the bare electrode is characterized by a large semi-circle in the high–medium frequency range, attributed to the charge transfer resistance (Rct) of the material. Its value is very large (36.5 kΩ), indicating that the transfer of electrons across the interface takes place with difficulty. The straight line at low frequencies is attributed to Warburg impedance (W) and describes the diffusion of the redox species within the double layer. The charging of the double layer due to the accumulation of ions from the solution at the interface is modelled by a constant phase element (CPE). In addition, the circuit contains the solution resistance (Rs). A similar electrical circuit was used to fit the data recorded with the graphene-modified electrode (see Figure S2 and the corresponding inset), but the Warburg impedance was replaced with another CPE due to the porous morphology of the surface. The Rct value obtained for the graphene-modified electrode was very low (9.91 Ω), proving the swift transfer of electrons across the interface. In addition, the real and imaginary values are considerably lower, which may indicate that there is a small amount of adsorbed molecules to hinder the electrochemical reaction.

Besides the determination of the graphene-modified electrode’s active area and charge transfer resistance, the next experiments were devoted to the investigation of the solution’s pH’s effect (pH 3.6–8.0) on the electrochemical response of the graphene-modified electrode towards CFX oxidation (10^−4^ M concentration). According to the literature [46], CFX is an ampholytic compound with pK_a_ values of 6.09 (carboxylic group) and 8.74 (nitrogen on the piperazinyl ring). Its isoelectric point (zwitterion) is at pH 7.4, and at slightly basic pH, CFX is very sensitive to photodegradation. The drug stability is higher in acidic solutions, where the basic nitrogen is fully protonated and the COOH group is not ionized. The stability of CFX in an acidic medium is important from a practical point of view, since the pH of liquid pharmaceutical formulations is generally acidic.

**Figure 6:**
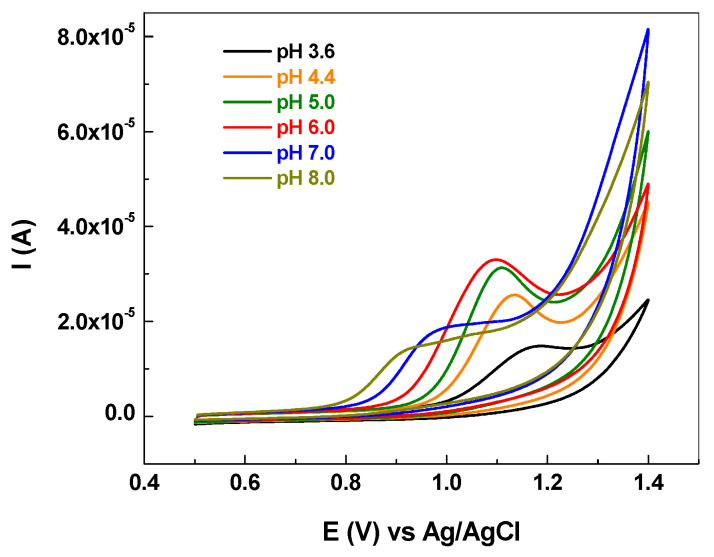
CVs recorded in buffer solutions of various pHs (from 3.6 to 8.0) in the presence of 10^−4^ M CFX; 10 mV/s scan rate.

**Figure 7:**
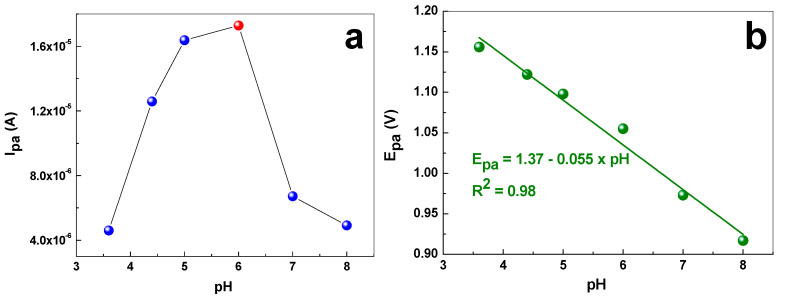
Variation of I_pa_ with the solution pH (**a**); variation of *E_pa_* with the solution pH (**b**).

The CV technique was applied to test the effect of the solution pH, since it provides information on both the anodic and cathodic processes. For this, acetate (pH 3.3, 4.4 and 5) and phosphate (pH 6, 7 and 8) buffers were employed. As can be seen in Figure 6, a well-defined oxidation peak appears in all acidic solutions (pH ≤ 6) which becomes very broad in neutral and alkaline conditions (pH 7 and pH 8). No peak is observed in the reverse scan, so the overall process is irreversible. The highest anodic peak current (I_pa_) was obtained in pH 6 PBS (Figure 7a); therefore, this solution was selected as the optimum electrolyte for the detection and quantification of CFX (in laboratory and real sample solutions).

The anodic peak potential (*E_pa_*) strongly shifts towards lower values with the pH increase, indicating the involvement of protons in the CFX electrochemical oxidation process (Figure 7b). The linear regression method that fits the experimental data follows the following equation: *E_pa_* = 1.37 − 0.055 × pH (R^2^ = 0.96). As can be observed, the slope value (55 mV/pH) is close to the theoretical value of 59 H^+^/n (mV/pH), so an equal number of protons and electrons are involved in the oxidation process of CFX (n is the number of electrons involved in the reaction).

**Figure 8:**
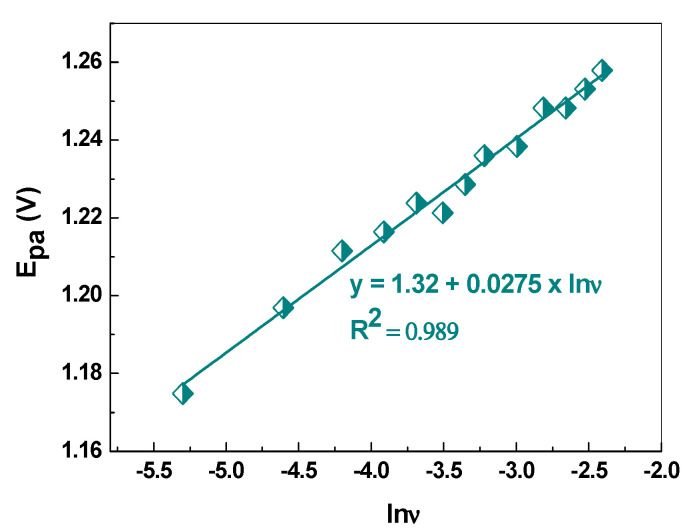
The variation of E_p_ with the natural logarithm of scan rate, lnυ, for the EGr/GC electrode.

Since the CFX oxidation at the surface of EGr/GC modified electrodes is an irreversible process, the Laviron equation [47] may be applied to determine the number of electrons involved in the oxidation (LSV technique). According to Laviron, *E_pa_* varies with the natural logarithm of the scan rate, ln*υ*, (Figure 8) as described by Equation (2):

(2) $$E_{pa}={E^{0}}^{\prime }-\frac{RT}{{(1-\alpha }_{a})nF}\ln\frac{RTk_{s}}{{(1-\alpha }_{a})nF}+\;\frac{RT}{{(1-\alpha }_{a})nF}ln\upsilon$$

where *α_a_* is the charge transfer coefficient, *k_s_* is the standard rate constant of the surface reaction, *n* is the number of electrons involved in the reaction and *E*^0′^ is the formal potential.

**Scheme 1:**
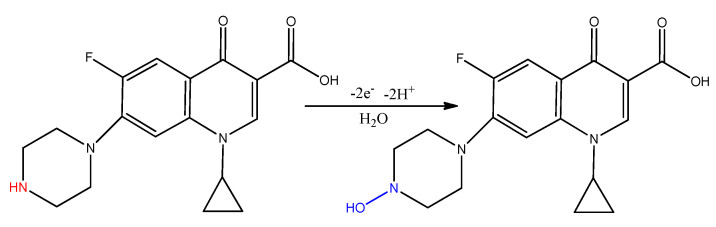
Electrochemical oxidation of CFX at EGr/GC electrode surface in pH 6.0 PBS electrolyte.

LSVs were recorded at various scanning rates (ranging between 2–100 mV/s) in pH 6.0 PBS solution containing 10^−4^ M CFX. From the linear regression equation (*E_pa_* = 1.32 + 0.0275 × ln*υ*), the value of (1 − *α_a_*)*n* was determined to be 0.93 (~1). Since for a totally irreversible electrode process *α_a_* is considered to be ~0.5, the number of electron transfers taking place during the oxidation of CFX is equal to two. Therefore, a total of two protons are also involved in the process. Based on the above findings and in good agreement with the literature, the electrochemical oxidation process of CFX at the surface of a EGr/GC electrode in pH 6.0 electrolyte is an adsorption-controlled process and follows the oxidation mechanism depicted in Scheme 1.

**Figure 9:**
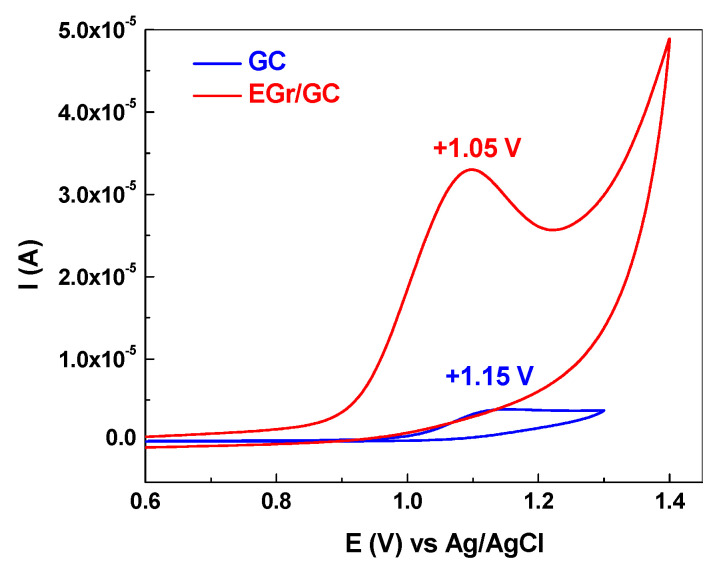
CVs recorded in 10^−4^ M CFX in pH 6.0 PBS electrolyte, with bare GC (blue) and EGr/GC (red) electrodes; 10 mV/s scan rate.

The advantages of employing graphene-modified electrodes are multiple, such as the considerable increase of the electrodes’ active areas, as well as the increase of the oxidation current along with the decrease of the oxidation potential. These can be clearly observed in Figure 9, where the oxidation peak recorded with the EGr/GC electrode is six times higher than that recorded with bare GC. In addition, the peak potential was shifted towards lower values, from +1.15 to 1.05 V, proving the electro-catalytic effect of graphene flakes attached to the GC surface.

**Figure 10:**
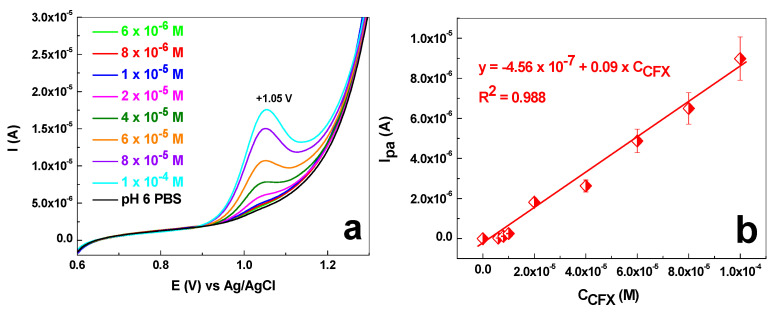
LSVs recorded in pH 6 PBS containing increasing concentrations of CFX, from 6 × 10^−6^ to 1 × 10^−4^ M; 10 mV/s scan rate (**a**); the corresponding calibration curve, with the linear regression equation (**b**).

In order to evaluate the analytical performance of the EGr/GC electrode, it was further tested using the LSV technique in solutions containing various CFX concentrations, from 6 × 10^−6^ to 1 × 10^−4^ M (10 mV/s scan rate). In Figure 10a the recorded LSVs are presented, while in Figure 10b the corresponding calibration plot and the linear regression equation, which describes the plot I_pa_ = −4.56 × 10^−7^ + 0.09 × C_CFX_ (R^2^ = 0.988), are presented. Based on the obtained experimental data, the analytical parameters were linear, ranging from 0.6 × 10^−7^ to 1 × 10^−4^ M CFX; the sensitivity was 0.09 A/M; the limit of quantification (LOQ) was 0.6 × 10^−7^ M; and the limit of detection (LOD) was 0.182 × 10^−7^ M (LOD was calculated by dividing LOQ by 3.3).

**Figure 11:**
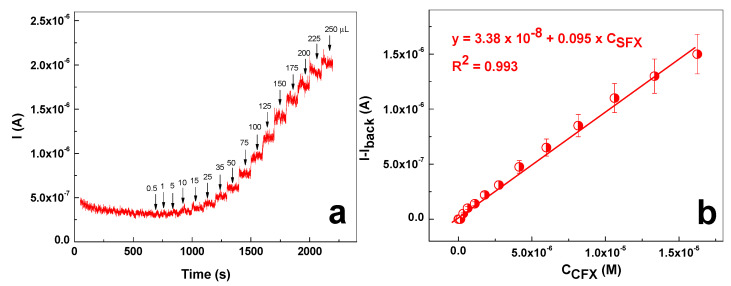
The chronoamperometric signal recorded in pH 6.0 PBS with the EGr/GC electrode after the addition of CFX aliquots from a 1 × 10^−4^ M stock solution; +1.1 V applied potential (**a**); the calibration plot obtained with background subtracted signals (I-I_back_) and the corresponding linear regression equation (**b**).

By employing chronoamperometry, better results were obtained, due to the fact that in this technique the measurement takes place in the diffusion layer at the working electrode surface. According to the IUPAC definition [48], the diffusion layer is the surrounding region of the electrode surface where the analyte concentrations are different from their value in the bulk solution. When the oxidation potential is applied to the working electrode, the analyte’s local concentration falls to zero, but the occurring gradient in the diffusion layer supplies the analyte from the bulk solution to the electrode surface. The recorded chronoamperogram and the corresponding calibration plot are presented in Figure 11a,b. In this case, the linear range was from 0.33 × 10^−8^ to 2.5 × 10^−5^ M CFX, the sensitivity was 0.095 A/M, the limit of quantification was 0.33 × 10^−8^ M and the limit of detection was 0.1 × 10^−8^ M. When plotting the calibration curve, background subtracted signals were employed (I_back_ = 0.3 × 10^−8^ A). As can be observed, the obtained analytical parameters of the EGr/GC modified electrodes determined through chronoamperometry are superior to those obtained with the LSV technique. As one can see in Figure 11b, there is a small change in linearity above the concentration of 10^−6^ M, which may be attributed the adsorption of CFX molecules at the graphene surface. CFX is a bulky molecule with three aromatic rings that can easily interact by non-covalent bonding (π-π interaction) with the graphene surface. Although such interactions are generally weak, the accumulation of CFX on graphene may occur, leading to the diminishing of the active surface. In consequence, the electrochemical signal is slightly decreased at higher concentrations.

The electrochemical performances of the EGr/GC electrode (linear range and limit of detection) were compared with previous results reported in the literature (see Table 2). As shown, the developed graphene-based sensor shows comparable, or even better, results in relation to other published protocols, providing clear evidence of its practical potential.

**Figure 12:**
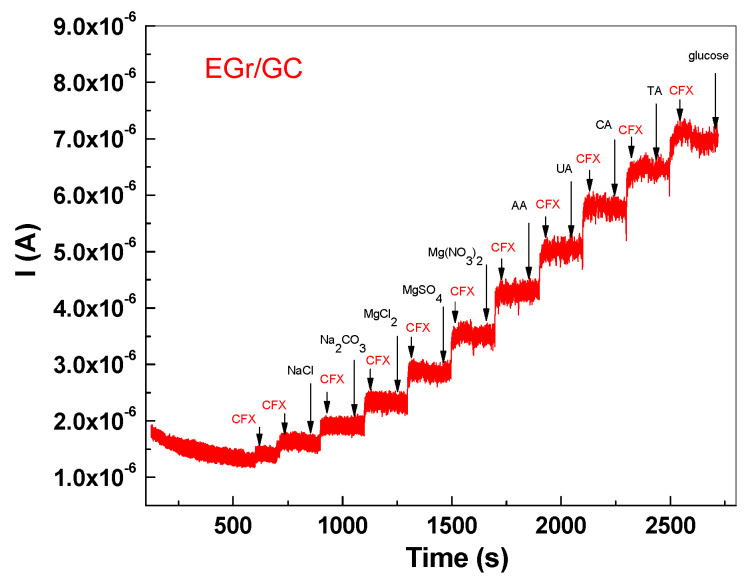
The chronoamperometric signal recorded in pH 6.0 PBS with the EGr/GC electrode after the addition of CFX aliquots from a 1 × 10^−4^ M stock solution and various interfering species (1 × 10^−3^ M stock solutions for the interfering species); +1.1 V applied potential.

The selectivity of the EGr/GC electrode towards CFX detection was tested in the presence of various inorganic and organic interfering species, such as NaCl, Na_2_CO_3_, MgCl_2_, MgSO_4_, Mg(NO_3_)_2_, ascorbic acid (AA), uric acid (UA), citric acid (CA), tartaric acid (TA) and glucose. The testing was performed since CFX may be found along with many other non-targeted species in domestic and industrial wastewater and pharmaceutical formulations. The ions and molecules may influence the detection process not only by altering the conductivity of the solution, but also by adsorption onto the modified electrode’s surface, limiting the sensor’s applicability for on-site monitoring. The chronoamperometric signal was recorded at a potential of +1.1 V in pH 6.0 PBS (5 mL initial volume) and each interfering species (from 1 × 10^−3^ M stock solutions) was successively added after CFX (from 1 × 10^−4^ M stock solution). As one can see in Figure 12, the majority of them had no effect on the electrochemical response of CFX, with the exception of glucose, which slightly decreased the signal.

Furthermore, the selectivity of the EGr/GC electrode was tested in presence of different antibiotics (TMP—Trimethoprim; SDZ—Sulfadiazine; SMZ—Sulfamethazine; SFX—Sulfamethoxazole; AZT—Azithromycin; TTC—Tetracycline; PABA—4-Aminobenzoic acid), as depicted in Figure S3 of the Electronic Supplementary Materials. As shown, TMP and AMP had no significant influence on the sensor’s response towards CFX detection, while two of the sulfonic antibiotics (SDZ and SFX), together with TTC, produce a slight decrease of the recorded chronoamperometric signal. At the same time, the interference of SMZ and PABA is translated into a slight increase of the recorded electrochemical signal. However, all the results confirm the excellent anti-interfering capacities and the good selectivity of the reported graphene-based sensitive substrate.

Due to the low interference, the developed graphene-modified electrode was considered a good candidate for CFX determination in real sample solutions. Hence, a pharmaceutical antibiotic drug (CIPRINOL) which contained ciprofloxacin as the active substance (250 mg/tablet) was bought from a local pharmacy. The tablet core contained other ingredients, such as povidone, sodium starch glycolate (type A), microcrystalline cellulose, colloidal anhydrous silicon dioxide, croscarmellose sodium and magnesium stearate. The tablet film was composed of hypromellose, talc, titanium dioxide (E171) and propylene glycol.

**Figure 13:**
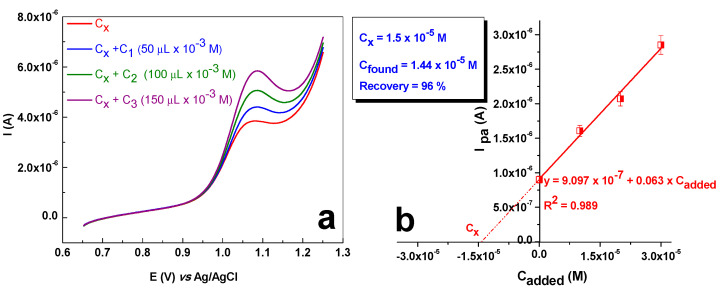
The CFX signal recorded using LSV, with the EGr/GC electrode in solutions containing CIPRINOL and spiked CFX from the stock solution, (1 × 10^−3^ M) pH 6.0 PBS supporting electrolyte; scan rate 10 mV/s (**a**); the standard addition curve obtained by plotting I_pa_ (A) versus the spiked CFX from the stock solution, C_added_ (M) (**b**).

First, the tablet (413.3 mg) was dissolved in pH 6.0 PBS (20 mL) resulting a stock solution of 3.77 × 10^−2^ M CFX. Next, 100 µL of the as-prepared stock solution was diluted to a total volume of 5 mL pH 6.0 PBS, resulting in a new solution with a concentration of 7.54 × 10^−4^ M CFX. Further, aliquots of 100 µL from this solution were transferred into four different beakers in which the maximum volume was set to be 5 mL. The first beaker, containing only the tablet solution in buffer (final concentration of 1.5 × 10^−5^ M), was regarded as the ‘unknown’ concentration, while the other three beakers were spiked with 50, 100 and 150 µL standard CFX solution, of 1 × 10^−3^ M concentration. LSV measurements were performed in each of the as-prepared solutions and the obtained peak current was plotted against the spiked CFX concentration (Figure 13a). The obtained calibration curve was used to determine the ‘unknown’ concentration from the real sample solution, resulting in 1.44 × 10^−5^ M (Figure 13b).

The applicability of the designed graphene-based substrate in simulated body fluids was also tested using artificial urine solution prepared according to the Shmaefsky protocol [65]. In short, aliquots of 100 µL CFX solution 1× 10^−4^ M were put into four different beakers. The first beaker was filled up to a maximum volume of 5.1 mL with artificial urine, resulting in a CFX concentration of 1.96 × 10^−6^ M; this was regarded as the ‘unknown concentration’ (C_x_). Next, the other three beakers were spiked with different amounts of CFX solution (50, 100 and 150 µL, respectively) from a stock CFX solution 1 × 10^−4^ M, and artificial urine was added up to the same maximum volume of 5.1 mL. Similar to the experiment performed in pharmaceutical CIPRINOL tablet solution, LSV measurements were performed on the as-prepared solutions and the results are depicted in Figure S4a of the Electronic Supplementary Materials. The peak intensity (I_pa_) was plotted against the spiked CFX concentration (C_added_)—please see Figure S4b—in order to determine the ‘unknown’ C_x_ concentration, resulting in an obtained value of 1.41 × 10^−6^ M. Thus, the calculated recovery is 71.9%, proving the potential of such sensoristic platforms for real applicability in the pharmaceutical and health sectors.

## 3. Materials and Methods

### 3.1. Chemicals

High-purity reagents were employed as provided. Ciprofloxacin (CFX, 98%) was purchased from Thermo Fisher Scientific (Waltham, MA, USA), while N,N-dimethylformamide (DMF); potassium hexacyanoferrate (III) (K_4_[Fe(CN)_6_] ≥99%); L(+)-Ascorbic acid (C_6_H_8_O_6_, ≥99%); D-(+)-glucose (C_6_H_12_O_6_, 99.5%); and citric acid (C_6_H_8_O_7_, ≥99.5%) were purchased from Merck (Darmstadt, Germany). Sodium sulphate (Na_2_SO_4_, 99.5%); boric acid (H_3_BO_3_, 99.5%); L-(+)-Tartaric acid (C_4_H_6_O_6_, ≥99.5%); potassium chloride (KCl, 99.98%); sodium chloride (NaCl, 99.98%); ammonium chloride (NH_4_Cl, 99.98%); calcium chloride dihydrate (CaCl_2_·2H_2_O, 99.98%); urea (CH_4_N_2_O, 99.0–100.5%); creatinine (C_4_H_7_N_3_O, >98%); sulfamethoxazole (C_10_H_11_N_3_O_3_S, ≥99.0%, SFX); trimethoprim (C_14_H_18_N_4_O_3_, ≥99.0%, TMP); and graphite rods (6 mm diameter, 99.995%) were acquired from Sigma-Aldrich (Taufkirchen, Germany). Alfa Aesar (Karlsruhe, Germany) provided uric acid (C_5_H4N_4_O_3_, 99%); potassium dihydrogen phosphate (KH_2_PO_4_, 99.99%); and methanol HPLC grade (CH_3_OH). Magnesium dichloride (MgCl_2_, 99.98%); magnesium sulphate (MgSO_4_, 99%); magnesium nitrate (Mg(NO_3_)_2_, 99.99%); and sodium carbonate (Na_2_CO_3_, 99.98%) were bought from REACTIVUL Bucuresti (București, Romania). VWR Chemicals (Leuven, Belgium) was the provider of sodium dihydrogen phosphate (H_2_NaO_4_P, 100%) and di-sodium hydrogen phosphate anhydrous (HNa_2_O_4_P, 99.7%). Sulfadiazine (SDZ, 98%); Sulfamethazine (SMZ, 99%); Ciprofloxacin (CFX, 98%); Tetracycline (TTC, 98%); Azithromycin (AZT, 99%); and 4-Aminobenzoic acid (PABA, 99%) were obtained from Thermo Fisher Scientific (Waltham, MA, USA). All working solutions were prepared in ultrapure water (Milli-QTM system, 18.2 MΩ × cm at 25 °C), kept away from direct sunlight exposure and refrigerated at 4 °C when not in use.

### 3.2. Apparatus

For the SEM characterization of the graphene sample, we employed a Hitachi HD2700 instrument (Hitachi, Tokyo, Japan) equipped with a cold field emission gun (CSEG). X-ray powder diffraction (XRD) technique allowed the structural characterization of graphene and was performed with a Bruker D8 Advance Diffractometer (40 kV; 0.5 mA) equipped with a LYNXEYE detector (λ = 1.5406 Å). The graphene Raman spectrum was recorded with an NTEGRA Spectra platform, placed on a NEWPORT RS4000 optical table and equipped with a SOLAR TII confocal Raman spectrometer coupled with an Olympus IX71 microscope in two different configurations (Moscow, Russia). After the electrochemical exfoliation of the graphite rods, the synthesized material was dried by lyophilization with a Christ-Alpha 1-4 LSC freeze-drier (Martin Christ Gefriertrocknungsanlagen GmbH, Osterode am Harz, Germany).

A SPECORD 250 PLUS spectrophotometer (Analytik Jena GmbH, Jena, Germany), in the 200–800 nm spectral range, was employed for the UV–Vis analysis. The FTIR spectrum measurement (4000–400 cm^−1^ spectral domain) was obtained with a resolution of 4 cm^−1^ using a JASCO 6100 FTIR spectrometer (KBr pellet technique). Around 1 mg of graphene was mixed with ~200 mg KBr and ground in an agate mortar. The ground mixture was pressed into a pellet; then, the spectrum was immediately recorded.

X-Ray Photoelectron Spectroscopy (XPS) measurements were performed under irradiation with an Al_Kα_ X-ray source (1486.6 eV) operated at 200 W, using a SPECS spectrometer equipped with a dual-anode X-ray source Al/Mg, a PHOIBOS 150 2DCCD hemispherical energy analyser and a multi-channeltron detector. The XPS survey spectra were recorded at 30 eV pass energy, 0.5 eV/step. The high-resolution spectra for individual elements were recorded by accumulating 15–20 scans at 30 eV pass energy and 0.1 eV/step. The surface cleaning was ensured through argon ion bombardment at 500 V for 5 min. The sample did not show electrostatic charging; thus, the binding energies are presented without any correction. Data analysis and experimental curve fitting of the *C 1s*, *O 1s* and *S 2p* spectra were performed using Casa XPS software, version 2.3.16 (Casa Software Ltd., Wilmslow, Cheshire, UK), with a Gaussian–Lorentzian product function and a non-linear Shirley background correction.

Various electrochemical techniques were employed for testing the bare (GC) and graphene-modified electrodes (EGr/GC), such as Cyclic Voltammetry (CV), Linear Sweep Voltammetry (LSV) and Chronoamperometry (CA). For this purpose, a Potentiostat/Galvanostat instrument, PGSTAT-302N (Metrohm-Autolab B.V., Utrecht, The Netherlands), coupled with a personal computer, was employed. The working electrode was either the bare glassy carbon electrode (3 mm diameter) or the electrode modified with graphene; the reference was an Ag/AgCl electrode; and the counter electrode was a large platinum sheet (2 cm^2^ area).

### 3.3. Exfoliation of Graphite Rods for Graphene Synthesis (EGr)

Two graphite rods connected to the exfoliation system (home-made) were employed as the anode and cathode in an electrochemical cell, previously filled with 0.05M sodium sulphate (Na_2_SO_4_), 0.05 M boric acid (H_3_BO_3_) and 0.05 M sodium chloride (NaCl). Before starting the graphene synthesis, the working parameters of the exfoliation system were set up as follows: the applied bias was 12 V; the current pulse duration was 0.8 s; and the pause between two pulses was 0.2 s [66]. The exfoliation process lasted for 4 h and it started shortly at the anode after the bias was applied. The sample deposited at the bottom of the cell was collected and washed by decantation with 10 L of distilled water. Next, the sample was dispersed in 100 mL distilled water and treated for 30 min in an ultrasound bath. Following this, for the removal of large graphite flakes, the material was filtered with Whatman qualitative paper (white ribbon filter) and finally dried by lyophilization. The sample was denoted EGr.

### 3.4. Modification of Glassy Carbon Electrode Surface with Graphene (EGr/GC)

N,N-dimethylformamide (DMF) was selected for the dispersion of the graphene sample (2 mg/mL) and aliquots of 0.5 µL of this colloidal suspension were drop-casted in successive layers on the GC surface. Due to its high boiling point (153 °C), DMF slowly evaporates at room temperature, so the graphene flakes may interact with the glassy carbon surface, forming a stable layer. After testing, the electrochemical signal of several electrodes covered with different volumes (5; 8; 10; 12 and 14 µL) of graphene/DMF solution towards 1 × 10^−4^ M CFX in pH 6 acetate buffer solution; the electrode covered with 10 µL was chosen as the best one. Following electrode modification, the electrochemical performances of EGr/GC and GC towards CFX detection and quantification were tested and compared.

## 4. Conclusions

In this work, a novel electrochemical sensor—based on glassy carbon surface modification with graphene, prepared through the electrochemical exfoliation of graphite rods in pulses of current in the presence of 0.05 sodium sulphate (Na_2_SO_4_), 0.05 M boric acid (H_3_BO_3_) and 0.05 M sodium chloride (NaCl)—was developed and tested for CFX detection. In addition to good stability and repeatability, the results showed that the presence of graphene at the sensor’s surface increases the sensitivity and the selective recognition of low levels of CFX in both laboratory solutions and real sample analysis. As a proof of concept, the developed sensor was successfully applied to CFX quantification in commercially available pharmaceutical formulations. All the evidence suggests that the proposed electrochemical sensor could make a good candidate with potential applicability in the clinical, pharmaceutical and environmental fields.

## Figures and Tables

**Table 1:** The structural parameters determined from the background subtracted pattern of the graphene sample.

Graphene Sample	2θ (degrees)	D (nm)	d (nm)	n	Amount (%)
FLG	21	1.1	0.433	3	86.4
MLG	26	10.46	0.34	30	13.6

**Table 2:** The linear ranges and limits of detection of various types of modified electrodes compared with those obtained in the present work from the EGr/GC electrode.

Electrode	Linear Range (M)	Detection Limit (M)	Technique	Reference
**rGO/PPR/GCE** rGO/PPR—reduced graphene oxide/poly(phenol red) GCE—glassy carbon electrode	0.2 × 10^−7^–4 × 10^−4^	0.2 × 10^−8^	DPV	[29]
**PEI@Fe_3_O_4_@CNTS/GCE** PEI@Fe_3_O_4_@CNTS—polyethylenimine@Fe_3_O_4_@carbon nanotubes GCE—glassy carbon electrode	0.3 × 10^−7^–7 × 10^−5^	0.3 × 10^−8^	DPV	[49]
**porous-Nafion-MWCNT/BDD** porous-Nafion-MWCNT—porous-Nafion-multiwall-carbon nanotubes BDD—boron-doped diamond	0.5 × 10^−8^–5 × 10^−4^ 0.5 × 10^−7^–1 × 10^−5^	0.05 × 10^−7^	DPV	[50]
**DNA/GCE** DNA GCE—glassy carbon electrode	1.0 × 10^−6^–1.0 × 10^−5^	0.117 × 10^−6^	DPV	[51]
**MWCNTs/GCE** MWCNTs—multiwall carbon nanotubes GCE—glassy carbon electrode	3.0 × 10^−6^–1.2 × 10^−3^	0.9 × 10^−6^	LSV	[52]
**CRGO/GCE** CRGO—Chemically-reduced graphene oxide GCE—glassy carbon electrode	6 × 10^−6^–4 × 10^−5^	0.21 × 10^−6^	SWV	[53]
**MgFe_2_O_4_/MWCNTs/GCE** MgFe_2_O_4_-MWCNTs—MgFe_2_O_4_ nanoparticles in modified multiwall carbon nanotubes GCE—glassy carbon electrode	0.1 × 10^−6^–0.1 × 10^−4^	0.01 × 10^−6^	CV	[54]
**CdS QDs/GCE** CdS QDs—Cd-based quantum dots GCE—glassy carbon electrode	1.0 × 10^−7^–1.0 × 10^−5^	2.2 × 10^−8^	DPASV	[55]
**MWCNTs/GCE** MWCNTs—multiwall carbon nanotubes GCE—glassy carbon electrode	4.0 × 10^−5^–1.0 × 10^−3^	6.0 × 10^−6^	AMP	[56]
**Graphene/GCE**	1.0 × 10^−7^–1.0 × 10^−5^	5.9 × 10^−8^	ASV	[57]
**COOH-functionalized MWCNT/GCE** MWCNTs—multiwall carbon nanotubes GCE—glassy carbon electrode	1 × 10^−6^–1 × 10^−4^	0.9 × 10^−6^	SWV	[58]
**PANI-β-CD-MWCNT/GCE** PANI-β-CD-MWCNT—multi-walled carbon nanotubes with β–cyclodextrin incorporated in a polyaniline film GCE—glassy carbon electrode	1 × 10^−7^–8 × 10^−7^	0.5 × 10^−7^	CV	[59]
**Au NP-β-CD-rGO/GCE** Au NP-β-CD-rGO/GCE—gold nanoparticle-coated with β-cyclodextrin-functionalized reduced graphene oxide nanocomposite GCE—glassy carbon electrode	1 × 10^−8^–1.2× 10^−7^	0.27 × 10^−8^	DPV	[60]
**rGO/GCE** rGO—reduced graphene oxide GCE—glassy carbon electrode	1 × 10^−6^–1 × 10^−4^	0.1 × 10^−6^	Flow injection with AMP	[61]
**rGO/MIP/GCE** rGO/MIP—reduced graphene oxide/molecularly imprinted polymer GCE—glassy carbon electrode	1 × 10^−9^–1 × 10^−7^	0.9 × 10^−7^	DPV	[62]
**SDS-GR/GCE** SDS-GR—sodium dodecyl sulphate/graphene GCE—glassy carbon electrode	0.3 × 10^−6^–1 × 10^−4^	0.29 × 10^−7^	DPV	[63]
**GN–PAAS–Pd/GCE** GN–PAAS–Pd—graphene–sodium polyacrylate–Pd GCE—glassy carbon electrode	1.08 × 10^−7^–1.8 × 10^−4^	0.45 × 10^−7^	AMP	[64]
EGr/GC	0.6 × 10^−7^–1 × 10^−4^ 0.33 × 10^−8^–2.5 × 10^−5^	0.182 × 10^−7^ 0.1 × 10^−8^	LSV AMP	* current work

* DPASV—differential-pulse anodic stripping voltammetry; ASV—anodic stripping voltammetry.

## Data Availability

Data will be provided upon reasonable request to the corresponding author.

## Supplementary Materials

The following supporting information can be downloaded at: https://www.mdpi.com/article/10.3390/ijms25073691/s1.
