# Supplementary material for: Harnessing Graphene-Modified Electrode Sensitivity for Enhanced Ciprofloxacin Detection
Source: Int J Mol Sci. 2024 Mar 26;25(7):3691. doi: 10.3390/ijms25073691 (PMC11012167; doi:10.3390/ijms25073691)
Supplement: Supplementary file 1 [file ijms-25-03691-s001.zip › ijms-2903473-supplementary.pdf]

Supporting Information Materials

Harnessing Graphene-Modified Electrode Sensitivity for Enhanced Ciprofloxacin Detection

Lidia Măgeruşan *, Florina Pogăcean, Bogdan-Ionuţ Cozar, Septimiu-Cassian Tripon and Stela Pruneanu *

National Institute for Research and Development of Isotopic and Molecular Technologies, Donat Street, 67-103 Cluj-Napoca, Romania
*Correspondence: lidia.magerusan@itim-cj.ro (L.M.); stela.pruneanu@itim-cj.ro (S.P.)

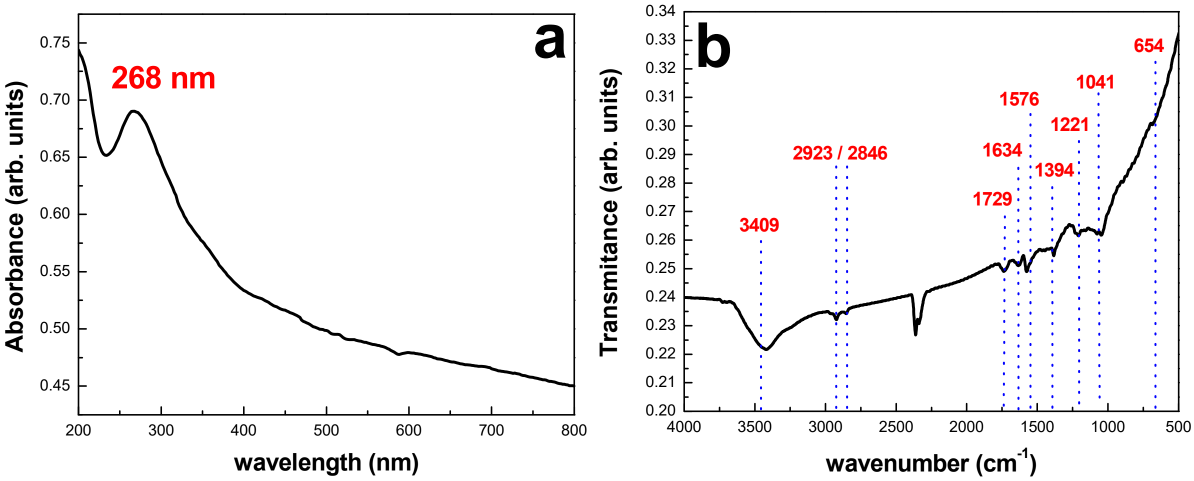

Figure S1. UV-Vis (a) and FTIR (b) spectra of exfoliated graphene sample.

Table S1. Assignments of each deconvoluted XPS peak based on their binding energies (BE) and atomic concentrations [AC, %], and full width at half maximum (FWHM) for each component.

	Binding energy (eV) [AC, %]	FWHM (eV)	Assignments
C1s	284.34 eV; 34.99%	1.53	sp^2 C=C /CH_n
	285.61 eV; 22.53%	1.19	sp3 C-C / CHn
	286.31 eV; 13.7%	1.21	C-O
	287.12 eV; 17.64%	1.67	C=O
	288.33eV; 6.18%	1.59	OH-C=O / COOH
	289.61 eV; 4.95%	1.72	$\pi \rightarrow \pi^*$ shake up satellite
O 1s	531.25 eV; 23.07%	2.48	C-O
	532.98 eV; 70.32%	2.59	OH-C=O / COOH
	536.05 eV; 6.61%	2.29	adsorbed H_2O

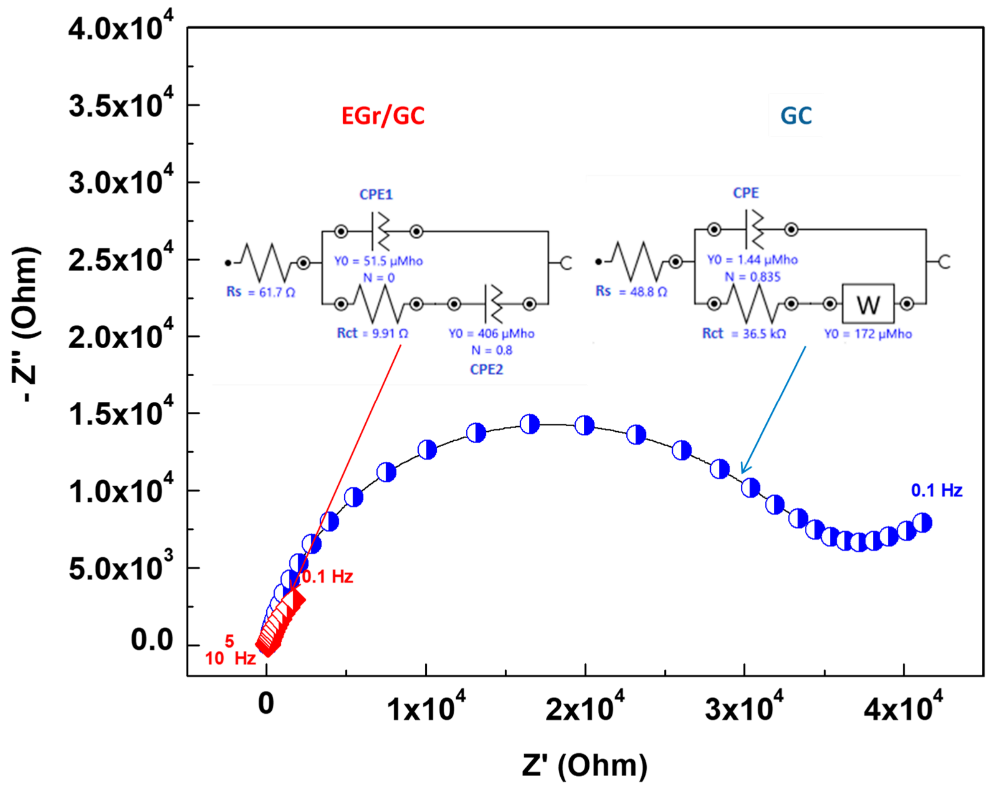

Figure S2. Nyquist plots recorded in 1mM potassium ferrocyanide (0.2 M KCl) for bare GC (blue) and graphene-modified electrode, EGr/GC (red); *inset:* the equivalent electrical circuits employed to fit the Nyquist plots of bare and graphene-modified electrode.

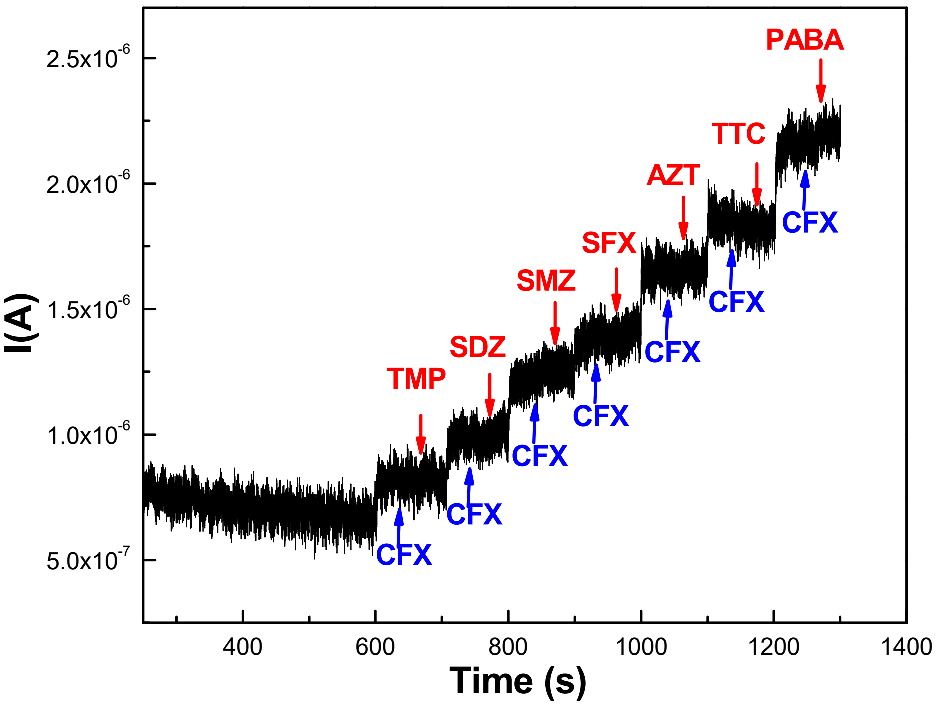

Figure S3. The amperogram recorded with EGr/GC electrode at +1.1V vs Ag/AgCl, during the successive addition of CFX and different antibiotics (TMP–Trimethoprim; SDZ–Sulfadiazine; SMZ–Sulfamethazine; SFX–Sulfamethoxazole; AZT–Azithromycin; TTC–Tetracycline; PABA - 4-Aminobenzoic acid); stirring: 300 rpm.

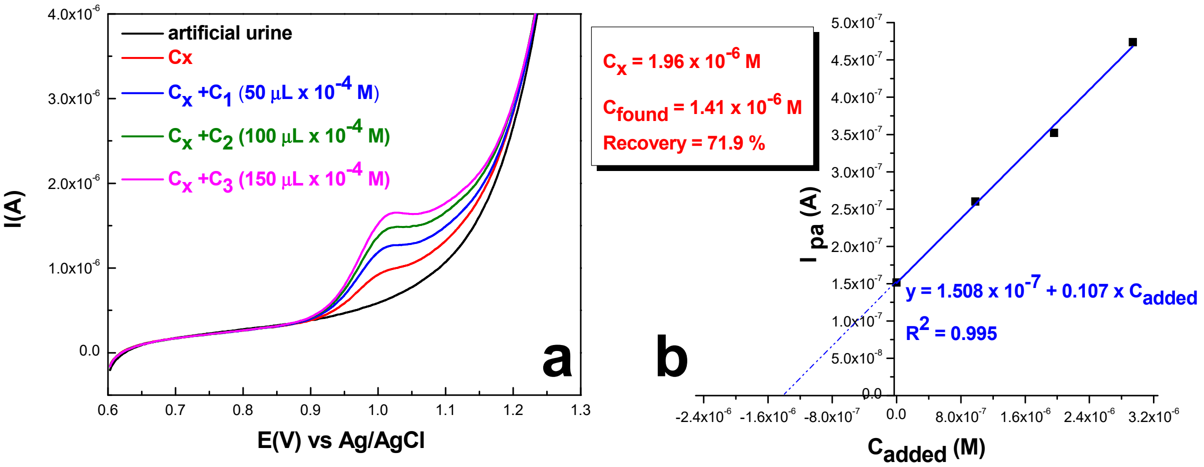

Figure S4. The LSV signal at the surface of EGr/GC electrode in artificial urine solution spiked with different amounts of CFX from the stock solution (1×10^{-4} M); scan rate 10 mV/s (a); The standard addition curve obtained by plotting Ipa (A) versus the spiked CFX from the stock solution - Cadded (M) (b).
